# Circadian-disruption-induced gene expression changes in rodent mammary tissues

**DOI:** 10.18632/oncoscience.292

**Published:** 2016-02-12

**Authors:** David Z. Kochan, Yaroslav Ilnytskyy, Andrey Golubov, Scott H. Deibel, Robert J. McDonald, Olga Kovalchuk

**Affiliations:** ^1^ Department of Biological Sciences, University of Lethbridge, Lethbridge, AB, Canada; ^2^ Canadian Centre for Behavioural Neuroscience, Department of Neuroscience, University of Lethbridge, Lethbridge, AB, Canada

**Keywords:** circadian disruption, breast cancer, transcriptome

## Abstract

Evidence is mounting that circadian disruption (CD) is a potential carcinogen in breast cancer development. However, despite the growing concern, to our knowledge, no studies have attempted a genome-wide analysis of CD-induced gene expression changes in mammary tissues. Using a rodent model system, a proven photoperiod-shifting paradigm, varying degrees of CD, and Illumina sequencing, we performed an exploratory genome-wide mRNA analysis in mammary tissues. Even though our analysis did not identify any significant patterns in mRNA levels based on the degree of CD, and the majority of groups did not show changes in gene expression on a large-scale, one group (two-week chronic ZT19) displayed 196 differentially expressed genes, 51 of which have been linked to breast cancer. Through gene-specific pathway analysis, the data illustrate that CD may promote breast cancer development through downregulation of DNA repair and p53 signaling pathways, thus promoting genomic instability and cancer development. Although these results have to be interpreted with caution because only a single group illustrated drastic changes in transcript levels, they indicate that chronic CD may directly induce changes in gene expression on a large-scale with potentially malignant consequences.

## INTRODUCTION

One of the most abundant malignancies in the world, breast cancer is already a serious public concern, and evidence is mounting that circadian disruption (CD) is a carcinogen that can trigger and promote the development of breast cancer [1-3]. Although numerous studies have been conducted on CD-induced breast cancer, the majority of these studies have focused on gene-specific analysis and very few studies have investigated the effect of CD-induced changes across a broad range of genes. As a consequence, the direct effect of circadian disruption in mammary tissues on important cellular mechanisms and pathways, such as DNA damage response (DDR) and p53 mediated signaling, remains largely unknown.

The DNA in every human cell is exposed to tens of thousands of aberrations per day, with stresses originating both endogenously and exogenously [4, 5]. Given this constant pressure, cells have evolved mechanisms to detect DNA lesions and to initiate repair of these lesions through signal pathways, with these processes being collectively called the DNA damage response [4]. DDR is a key mechanism to ensure proper cellular function, and related pathways are frequently deregulated in cancers [4]. Although changes to DNA repair pathways in tumorigenesis can vary greatly depending on different factors, such as the type of breast cancer and the stage of development, in terms of tumour initiation, downregulation of DNA repair has been concretely linked to increased genomic instability and progression of cancer [6-8].

Linked to the DDR system as a potential failsafe mechanism by initiating programmed cell death, apoptosis helps to maintain proper cell function and proliferation [9, 10]. Extensive research has shown that decreased levels of apoptosis is a hallmark of cancer progression, with aberrant activity of pro-apoptotic and anti-apoptotic genes playing a crucial role in this process [10]. Amongst the most important pro-apoptotic genes is p53, a corner stone of tumour suppression activity that has been described as the guardian of the genome because it responds to various stress signals and helps maintain proper cellular function [11, 12]. Influencing numerous cancer-relevant pathways besides apoptosis, the p53 signaling pathway is often aberrantly regulated in many malignancies, making it a crucial player in cancer development.

Although numerous studies have investigated links between circadian disruption and breast cancer, to our knowledge, no studies have investigated CD-induced changes on gene expression profiles in mammary tissues. The only studies to investigate gene expression in mammary tissues in relation to circadian rhythms, focused on the influence of mammary development or chemical induction on specific circadian genes within the mammary gland [13, 14]. In fact, the studies that have come closest to investigating broad CD-induced gene expression profiles in mammary tissues have been DNA methylation studies on shift worker blood samples [15-17]. Since epigenetic profiles of peripheral blood samples have shown a strong correlation with reflective changes in mammary tissues, these DNA methylation profiles may correlate to mammary gland transcript levels [18, 19]. However, these studies still do not represent a direct attempt to investigate CD-induced gene expression profiles in mammary tissues, and provide no information on crucial pathways and cellular mechanisms such as DDR and p53 mediated signaling. Therefore, the current study employed a proven photoperiod-shifting paradigm on a rodent model system, to investigate the effect of varying degrees of circadian disruption on wide-scale gene expression in mammary tissues.

## RESULTS

### Circadian disruption results in the differential expression of a broad range of genes linked to breast cancer

**Figure 1:**
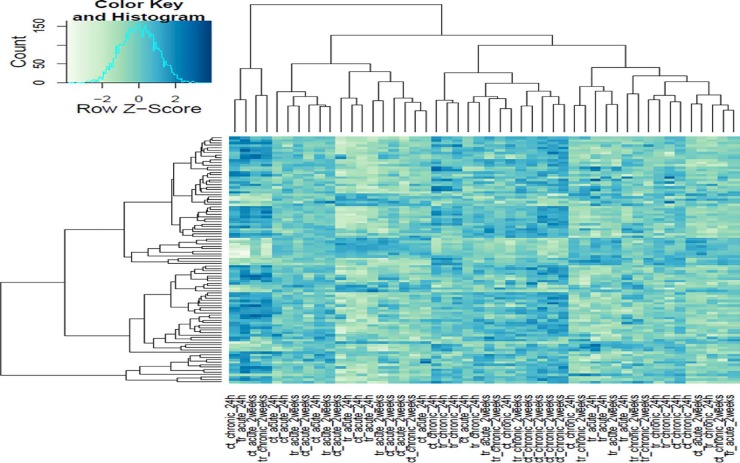
Heat map cluster of the Illumina gene sequencing results All of the sequenced samples based on the degree of circadian disruption and tissue extraction times.

**Figure 2:**
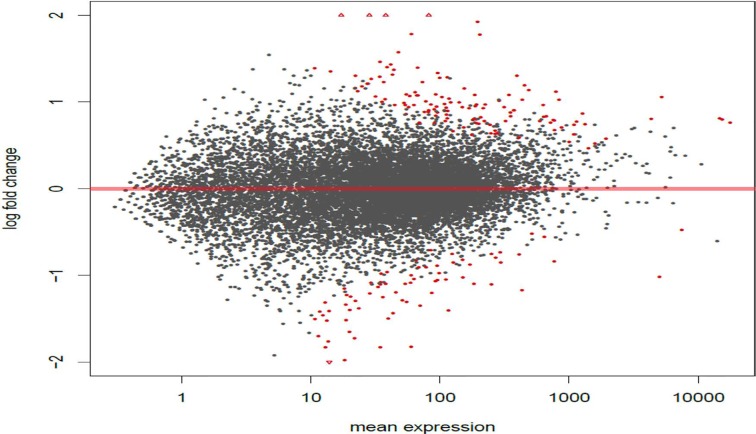
Circadian disruption causes large scale gene expression changes in the 2-week chronic ZT19 group MA plot based on the gene expression Illumina sequencing results for the 2-week chronic ZT19 group. The y-axis represents log fold change, while the x-axis represents mean expression. The plots represent all the gene expressions that were identified, and the red plots represent genes that showed a significant expression difference; P-adjusted < 0.1, N=6.

In this study, we investigated the effect of varying degrees of circadian disruption on gene expression in the mammary tissues of Sprague Dawley rats. The influence of light dependent Zeitgeber times was also incorporated to investigate possible fluctuations within a 24-hour circadian cycle. There were no significant patterns in gene expression between the various groups with regards to the degree of CD, tissue extraction time after CD, or ZT (Figure 1, Table 1). Although the sequencing results identified a broad range of differentially expressed genes, many of which are linked to breast cancer, only two groups illustrated more than ten changes in gene expression, the 24-hour acute ZT06 and two-week chronic ZT19 groups (Table 1). The majority of these differences were mainly found in the two-week chronic ZT19 group, in which 196 differentially expressed genes were identified, 51 of which have been linked to breast cancer (Table 1; Figure 2).

**Table 1:** Circadian disruption induces changes in gene expression

Group	Number of Genes Differentially Expressed	Number of Genes linked to Breast Cancer
24-hr Acute ZT06	15	12
24-hr Acute ZT19	0	0
2-Week Acute ZT06	0	0
2-Week Acute ZT19	2	2
24-hr Chronic ZT06	2	0
24-hr Chronic ZT19	5	4
2-Week Chronic ZT06	4	3
**2-Week Chronic ZT19**	**196**	**51**

Number of genes differentially expressed in all the experimental groups based on the Illumina sequencing data.

### The CD-induced gene expression changes in the two-week chronic ZT19 group correlate to disturbances in breast cancer related pathways

**Figure 3:**
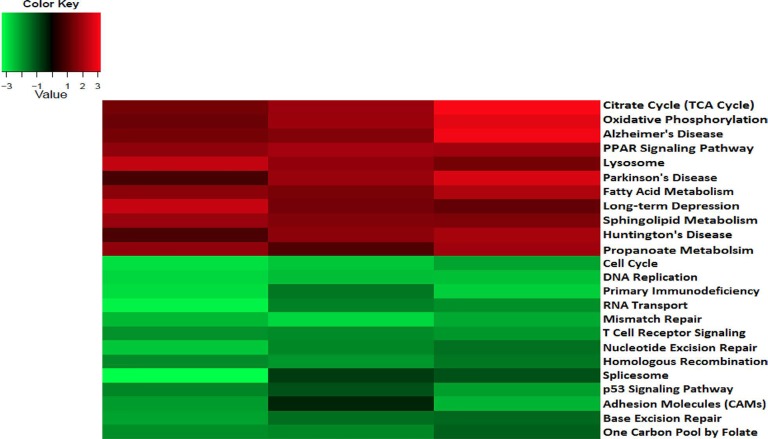
Circadian disruption causes changes to disease related and breast cancer relevant pathways in the 2-week chronic ZT19 group Heat map cluster based on the gene expression Illumina sequencing results for disease related and breast cancer relevant pathways in the 2-week chronic ZT19 group. The red bands represent over expressed pathways and the green bands represent under expressed pathways.

**Figure 4:**
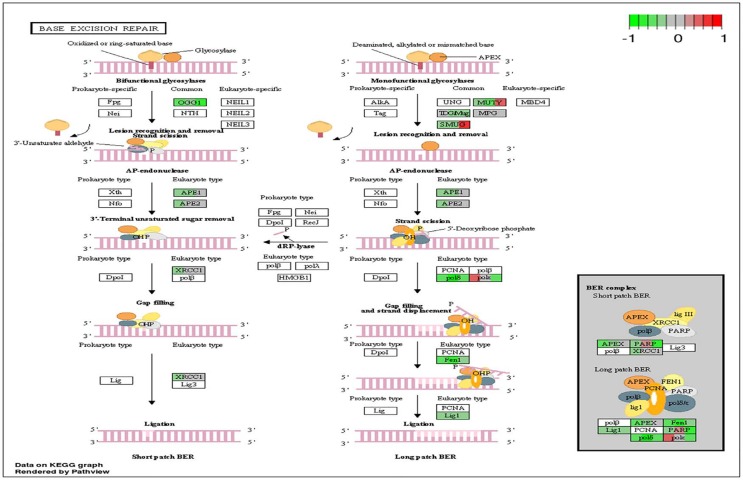
Circadian disruption induced changes to the base excision repair pathway Each gene is broken down into three portions and represents the three sequenced samples, with red correlating to overexpressed and green correlating to underexpressed genes. Pathways were generated using KEGG graphs and rendered by Pathview.

**Figure 5:**
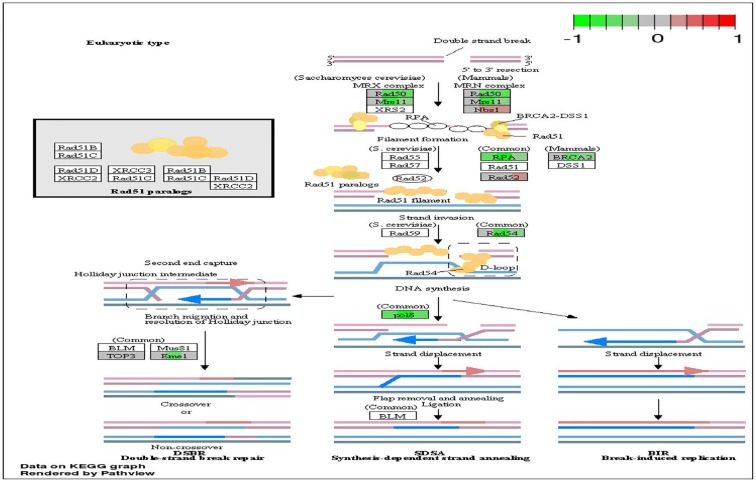
Circadian disruption induced changes to the homologous recombination pathway Each gene is broken down into three portions and represents the three sequenced samples, with red corresponding to overexpressed and green corresponding to underexpressed genes. Pathways were generated using KEGG graphs and rendered by Pathview.

**Figure 6:**
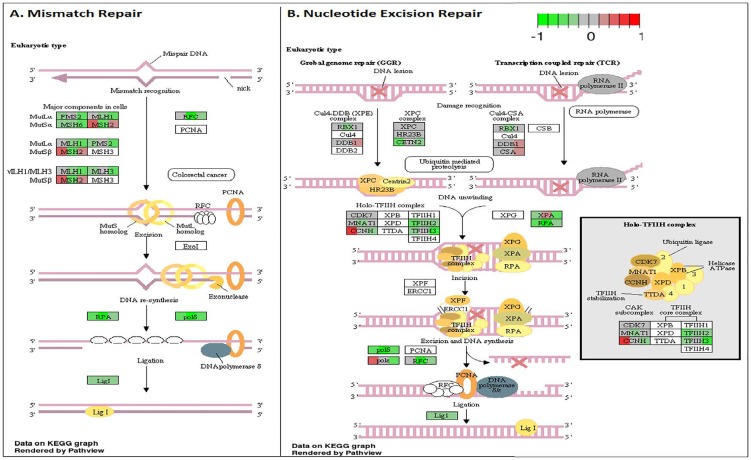
Circadian disruption induced changes to the mismatch repair and nucleotide excision repair pathways **A.** Mismatch Repair pathway and **B.** Nucleotide Excision Repair pathway based on the Illumina gene sequencing results. Each gene is broken down into three portions and represents the three sequenced samples, with red correlating to overexpressed and green correlating to underexpressed genes. Pathways were generated using KEGG graphs and rendered by Pathview.

**Figure 7:**
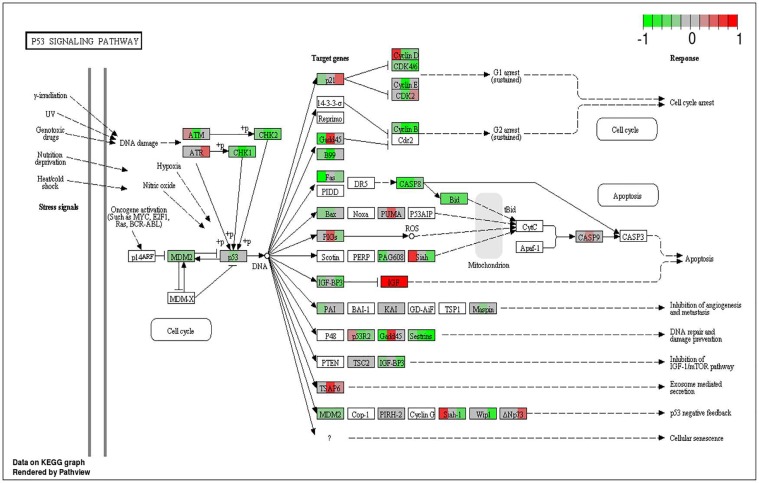
Circadian disruption induced changes to the p53 signaling pathway Each gene is broken down into three portions and represents the three sequenced samples, with red correlating to overexpressed and green correlating to underexpressed genes. Pathways were generated using KEGG graphs and rendered by Pathview.

To analyze the significance of the gene expression changes in the two-week chronic ZT19 group in terms of potential complications and breast cancer, pathway analysis was performed. The results illustrated that a substantial number of pathways and processes linked to diseases and complications were altered in these rats (Figure 3). Based on this data, gene-specific pathway analysis for some of the breast cancer-relevant pathways was performed to gain more details and insight into the effects of CD. The analysis revealed that numerous DNA repair pathways were altered: base excision repair (BER), homologous recombination (HR), mismatch repair (MMR), and nucleotide excision repair (NER) (Figure 4, Figure 5 and Figure 6). The overall gene expression patterns amongst these pathways correlated to decreased DNA repair. Gene-specific pathway analysis was also performed on the p53 signaling pathway, with the gene expression patterns correlating to decreased p53 signaling, decreased DNA repair, and decreased apoptosis (Figure 7).

### qRT-PCR validation produces the same expression trends, but not significant differences

**Figure 8:**
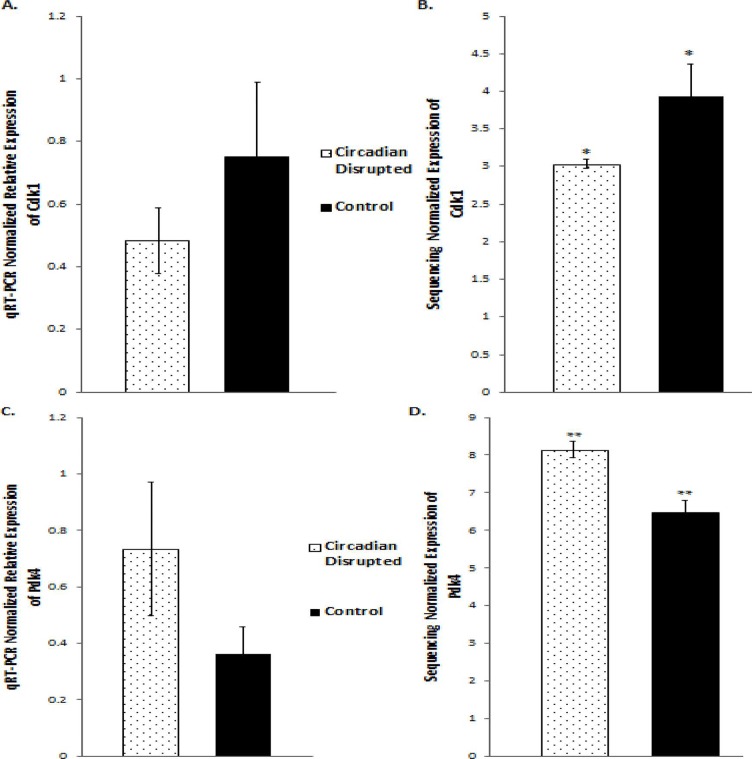
qRT-PCR and Illumina sequencing data for the *Cdk1* and *Pdk4* genes **A.** Mean relative expression of the *Cdk1* gene based on qRT-PCR, Controls (N=5), CD (N=5). **B.** Mean Relative expression of the *Cdk1* gene based on Illumina sequencing, Controls (N=3), CD (N=3), *p-adjusted < 0.05 **C.** Mean Relative expression of the *PDK4* gene based on qRT-PCR, Controls (N=5), CD (N=5). **C.** Mean relative expression of the *PDK4* gene based on Illumina sequencing, Controls (N=3), CD (N=3). **p-adjusted < 0.01. Error bars represent SEM.

**Figure 9:**
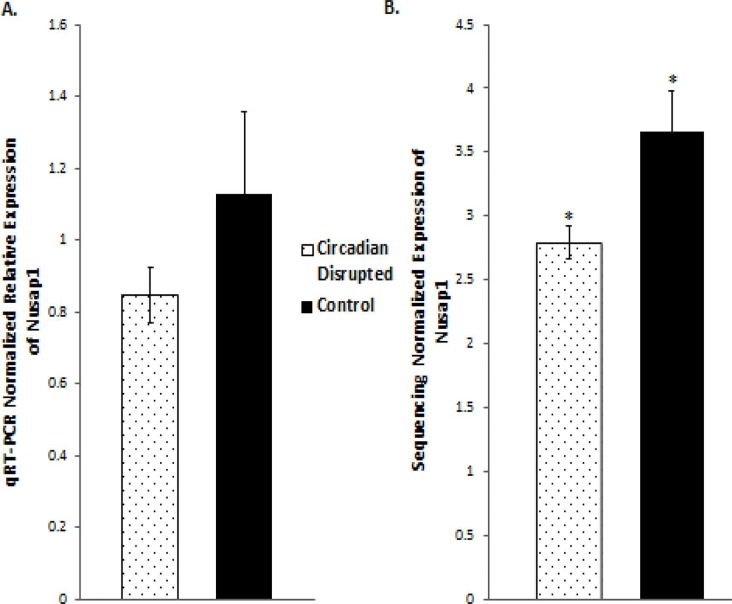
qRT-PCR and Illumina sequencing data for the *Nusap1* gene **A.** Mean relative expression of the *Nusap1* gene based on qRT-PCR, Controls (N=5), CD (N=5). **B.** Mean Relative expression of the *Nusap1* gene based on Illumina sequencing, Controls (N=5), CD (N=5), *p-adjusted < 0.05. Error bars represent SEM.

Validation of the sequencing results was performed on several of the differentially expressed genes through qRT-PCR analysis, including the *Cdk1*, *PDK4*, and *Nusap1* genes (Figure 8 and 9). Although the qRT-PCR data produced the same expression trends, the data did not produce significant differences (Figure 8 and 9). In general, the same pattern was observed in the majority of genes that were analyzed through qRT-PCR: similar expression trends, but no significant difference. These results may be due to the two samples that did not undergo Illumina sequencing usually producing qPCR expression trends that were not consistent with the sequencing data, while the three sequenced samples were consistent. Given that multiple reference genes with acceptable stability were used (Table 2), it is unlikely that the qRT-PCR data is unstable. This indicates that the gene expression differences based on Illumina sequencing in the two-week chronic ZT19 group may be due to the small sample size, which would explain such drastic expression differences in this experimental group (Table 1). However, the qPCR expression trends are consistent when all ten samples are analyzed, indicating that with a larger sample size, significant gene expression changes may have been reproduced through qRT-PCR.

**Table 2:** Stability data for the qRT-PCR results

**NormFinder**			
Gene Name	Stability Value	Best Combination of Two Genes	Combined Stability Value
ATP5b	0.027	ATP5b and Sdha	0.032
Sdha	0.058		
Tbp	0.115		
**GenNorm**			
Gene Name	Coefficient Variance		M-Value
ATP5b	0.0671		0.1929
Sdha	0.0666		0.1929

Normfinder stability values, and GeNorm coefficient and M-values for the reference genes used in the qRT-PCR analysis.

*Cut off for acceptable stability: CV < 0.25; M-Value < 0.5.

## DISCUSSION

Here we used a well-established rodent model to analyze circadian deregulation-induced breast cancer. Although rat strains differ in cancer susceptibility, such as Sprague Dawley rats being prone to spontaneous breast cancer, rats are excellent experimental models and have been used to study circadian regulation and breast carcinogensis [20, 21, 22]. Numerous publications have provided consistent evidence that circadian disruption acts as a carcinogen in breast cancer development, but no studies have conducted large-scale gene expression profiles on CD-induced changes in mammary tissues. Therefore, in this study we investigated the effect of varying degrees of CD on gene expression in rat mammary tissues. Although our findings did not produce any significant patterns based on the degree of CD or tissue extraction time, one group, the two-week chronic ZT19 group, did illustrate drastic changes in gene expression, thus indicating that long-term circadian disruption may be linked to a cascade of differential gene expression in mammary tissues (Figure 1 and 2; Table 1).

DNA repair mechanisms are key components of the DNA damage response, and the sequencing data illustrates that several DNA repair pathways were aberrantly expressed due to CD in the two-week chronic ZT19 group (Figure 3). Specifically, gene-specific pathway analysis showed that key genes in base excision repair (BER), homologous recombination (HR), mismatch repair (MMR), and nucleotide excision repair (NER) were down regulated (Figures 4–6). Decreased DNA repair has been associated with increased genomic instability and progression of cancer, and these results show that CD may decrease DNA repair on multiple fronts [8]. Furthermore, amongst these repair mechanisms, homologous recombination has been shown to play a crucial role in breast cancer development [23]. HR is involved in double strand break (DSB) repairs, and mutations to genes involved in this process are linked to tumours and gene rearrangements; HR can also be viewed as a last resort for DNA repair if DNA lesions are not identified and mended by other repair mechanisms [23]. Gene pathway analysis illustrates that CD-induced down regulation of HR was associated with decreased expression of *BRCA2*, a tumour suppressor gene that has been extensively linked to breast cancer development (Figure 5) [24]. Lowered expression of *Rad50* and *Mre11*, both of which are key components of the MRN complex and linked to increased breast cancer susceptibility, was also identified (Figure 5) [25]. In addition, sequencing results illustrate significant decreased expression of the *Nusap1* gene (Figure 9), which has been shown to regulate levels of *BRCA1*, an extensively studied tumour suppressor gene involved in double strand breaks and breast cancer [26]. These results indicate that CD-induced breast cancer development may be linked to aberrant DNA repair through multiple mechanisms, with silencing of important genes involved in homologous recombination possibly playing a crucial role.

Another pathway crucial to breast cancer development that is also linked to the DDR system is the extensively studied p53 signaling pathway, and our data shows that CD caused decreased activity in this pathway in the two-week chronic ZT19 group (Figure 3). The p53 pathway has long been a corner-stone of cancer research because of its important role in the cell cycle and apoptosis [11]. Gene expression pathway analysis illustrates that key genes in the p53 mediated signaling pathway were mostly down regulated, while the insulin-like growth factor (*IGF*) gene was up regulated (Figure 7). Amongst the most consistently down regulated genes were check point kinase 1 (*CHK1*), *CHK2*, *CASP8*, *Bid*, and *sestrins* (Figure 7). Both CHK1 and CHK2 play a crucial role in cell cycle arrest and apoptosis, are part of the DDR mechanism, and can activate p53 [4]. The *CASP8* and *Bid* genes are part of a signaling cascade that can induce apoptosis, with *CASP8* being able to modify *Bid* activity [27]. The *sestrin* genes are induced by p53 upon DNA damage, help regulate stress responses to environmental stimuli, and have been shown to decrease tumour growth in some cancer cells [28]. Finally, expression of *IGF* was up regulated in all three of the sequenced samples (Figure 7). IGF has been identified as an inhibitor of apoptosis in many different cell types through various mechanisms, including inactivation of *CASP8* through FLIP activity [27]. Taken together, these CD-induced gene expression patterns correlate with decreased apoptosis and increased genomic instability, indicating further contributions to cancer initiation and echoing the theme illustrated by the CD-induced changes in DNA repair mechanisms previously discussed.

Although the results discussed thus far have provided compounding evidence on the potential influence of CD on breast cancer development through aberrant gene expression patterns, only limited conclusions can be drawn because the changes are isolated to one group (Table 1). As mentioned previously, no patterns in gene expression changes were observed based on the degree of CD or tissue extraction times, with only the two-week chronic ZT19 group undergoing changes in gene expression on a large enough scale to significantly influence breast-cancer-relevant pathways (Table 1). Furthermore, although the qPCRs generated the same expression trends as the sequencing results; the trends were not significant based on the qPCR data (Figure 8 and 9). Given that the qPCR reference genes were stable (Table 2) and the qPCR data typically showed opposite expression trends for the non-sequenced samples, this indicates that the drastic expression trends identified in the two-week chronic ZT19 group may be due to small sample size or be a reflection of an inter-individual variability response to CD. However, the offsetting contribution of the non-sequenced samples to the qPCR results was not enough to alter the same expression trend (Figure 8 and 9). This indicates that with a larger sample size in every group, large-scale gene expression changes may have been produced in the other groups as well.

Our previous publication, which utilized the same rodent model, CD-scheme, and group sizes employed in this study, illustrated significant patterns of CD-induced miRNA expressions in mammary tissues based on the degree of CD and time of tissue extraction [1]. Therefore, the inconsistent results generated between the different groups in this study may not be due to the small sample size, but may instead hint at the complexity of cellular changes that are occurring without drastic changes in transcript levels. First, it is important to note that changes in miRNA levels do not have to correlate to changes in transcript levels because this relationship is based on the complementarity between the miRNA and its target gene [29]. In mammals, a single miRNA can target multiple genes with varying complementarity, and statistical analysis has shown that as little as 3.2% of miRNA-mRNA pairs illustrate a negative correlation [29, 30]. Second, in eukaryotes, ∼60% of the variation in protein levels cannot be explained by transcript levels, meaning that a variety of post-transcriptional mechanisms are influencing gene expression [31]. Based on these facts and our previous findings, this opens up the possibility of miRNAs being more prone to CD-induced changes in mammary tissues and playing a more prominent role in the initiation of CD-induced breast cancer.

Cancer is an age-associated disease, and its progression and development has been linked to the DNA damage theory of ageing [32, 33]. Since the 2-week chronic group represents the oldest rats in the study, the carcinogenicity of CD in terms of breast cancer development may be dependent on age, with CD triggering decreased DDR with increasing age. Therefore, molecular epigenetic mechanisms of circadian deregulation and carcinogenesis need to be further analyzed in an age domain, as recent studies have shown that circadian disruption accelerates aging and promotes tumorigenesis in rats, especially in older animals [21, 22, 34].

To our knowledge, this study represents the first attempt at directly investigating wide range CD-induced changes to gene expression in rodent mammary tissues. Although the results did not produce significant patterns or findings on the influence of circadian disruption based on the degree of CD or tissue extraction time, the study did provide evidence that chronic CD may directly induce gene expression changes on a significant scale in mammary tissues. Specifically, the results indicate that circadian disruption may alter DNA damage response mechanisms and p53 signaling in a manner that initiates and promotes breast cancer development. However, based on the results, it seems that the small sample size may be contributing to the generated results, or masking the extent of the CD-induced changes in gene expression. Therefore, repeating the experimental design with some slight variations and increased group size is a warranted future step.

In conclusion, although these results have to be interpreted with caution, they signify that chronic CD may induce potentially malignant changes to DNA damage response mechanisms and p53 signaling in mammary tissues. The role of circadian deregulation and its potential to exacerbate chemical-induced carcinogenesis also needs to be analyzed in detail in the future [35]. Many intracellular processes, such as those that control cellular levels of nicotinamide adenine dinucleotide (NAD(+)) are rhythmic and controlled by the circadian clock, and their alterations may contribute to carcinogensis [36]. Additionally, it would be important to dissect the link between CD-induced epigenetic deregulation and breast cancer in terms of novel diet and life style-based preventative strategies [35, 37].

## MATERIALS AND METHODS

### Animal model and circadian disruption paradigm

Female Sprague Dawley rats from Charles River (Quebec) were housed at the Canadian Center for Behavioural Neuroscience at the University of Lethbridge. The rats were housed in a sterile facility in a temperature controlled room, two per cage, and given food and water *ad libitum*. Handling and care of the animals was performed in accordance with the recommendations of the Canadian Council on Animal Care, and the procedures were approved by the University of Lethbridge Animal Welfare Committee. Before the start of the experiment, all the rats were entrained to a 12-hour light-dark cycle for 22 days to allow entrainment to a normal light schedule. At 83 days old, the rats were then randomly assigned to different treatment and control groups.

Circadian disruption was induced by following a photoperiod-shifting paradigm that has been shown to cause physiological and behavioural changes in rodents [38-41]. In total, 40 female rats underwent this photoperiod-shifting (PS) paradigm. To stimulate PS, the colony lights were turned on three hours earlier each day. To investigate the effect of varying degrees of CD, the 40 rats were separated into acute and chronic circadian disruption groups. Twenty rats underwent acute photo-period shifting, which consisted of lights coming on three hours earlier each day for a total cycle time of six days. Another 20 rats underwent chronic photoperiod-shifting, which consisted of a rotation between lights coming on three hours earlier each day for six days, and then ten days of a regular 12-hour light-dark cycle, for a total cycle time of 54 days. For both the acute (6 days) and chronic groups (54 days), following the PS cycle, the rats were exposed to a normal 12-hour light-dark cycle until it was time for tissue extractions.

The acute (20 rats) and chronic (20 rats) CD groups were then separated further based on the time of tissue extraction. Mammary tissue extractions occurred 24 hours and two weeks following acute or chronic circadian disruption, with ten rats from each CD group undergoing tissue extractions at each of these times. To account for and investigate the potential influence of specific time points within a 24-hour circadian cycle, two different tissue extraction time points, each corresponding to a specific Zeitgeber time, were performed on each tissue extraction day (24 hours and two weeks following CD). Half of the rats (five) in each tissue extraction group were sacrificed at ZT06 (6 hours after lights on) and the other remaining rats (five) from each group were sacrificed at ZT19 (19 hours after lights on). These two different ZT points were chosen because they represented the light and dark phases of the circadian cycle.

Both the acute (20 rats) and chronic (20) control groups were exposed to a 12-hour light-dark cycle for either six days (acute) or 54 days (chronic). The rats from each CD control group were then exposed to a 12 hour light-dark cycle for either 24 hours (10 rats) or two weeks (10 rats) depending on the time of tissue extraction for the corresponding experimental group. From each tissue extraction control group (10 rats), five rats were sacrificed at ZT06 and five rats were sacrificed at ZT19 on the corresponding tissue extraction day.

Euthanasia of the rats was performed through anesthesia with Isoflurane (4–5 %; oxygen at 2 liters per minute) and decapitation by a guillotine, with euthanasia of the rats alternating between the control and experimental rats. The mammary glands were collected, immediately stored in liquid nitrogen, and stored long-term at a temperature of −80°C.

### Total RNA extraction

Whole mammary tissues were ground in liquid nitrogen using sterile, chilled mortars and pestles. Approximately 0.05 g of ground tissue from each sample was then suspended in Zymo Research tri-reagent, and lysed using two cycles of the Qiagen Tissue Lyser II for 2 minutes at 25 Hz. Total RNA was then extracted using the direct-zol RNA Miniprep kit from Zymo research (R2053). The quality of the RNA was then checked using Nanodrop 2000c, and quality bio-analysis was conducted using the Agilent 2100 and the Agilent Small RNA Kit and Chip (5067-1548), with only samples having a RIN value greater than eight being used in downstream applications.

### Gene expression sequencing and bioinformatics analysis

Three samples from each tissue extraction and ZT group for both the experimental and control groups were randomly chosen to undergo sequencing analysis. The TruSeq RNA Sample Preparation Kit V-2/SetA from Illumina (RS-122-2001) was used to prepare small RNA libraries from 1 μg of total RNA. qPCRs were then run to validate the libraries following the PCR program recommended by the Illumina RNA Sample Prep Kit (3 mins at 95°C; 40 cycles of 3 sec. at 95°C, 30 sec. at 60°C). Cluster generation for sequencing was performed using cBot and the TruSeq SR Cluster Kit v2-cBot-GA (GD-300-2001) from Illumina. Single-read sequencing was performed using the TruSeq SBS Kit v5-GA (FC-104-5001) from Illumina on the Genome Analyzer GAIIx at 36 cycles.

Bioinformatics approaches on the sequencing data were used to define gene expression levels amongst the different experimental groups. Basecalling and demultiplexing were performed using CASAVA 1.8.1 pipeline (Illumina) with default settings. The quality of the libraries was evaluated using FastQC v0.10.1 software. Contaminating sequences (adapters, phiX, polyA, polyC, ribosomal RNA) were filtered out, and filtered sequence reads were aligned to the rat genome assembly Rnor 5.0 (Ensembl) from Illumina's iGENOME database. Files in sam format were converted to bam, sorted by chromosomal position, and indexed. Further quality control was performed using the RSeQC_2.3.7 software package, with no libraries being removed due to quality problems.

### qRT-PCRs

Validation of the sequencing results was performed by qRT-PCR. cDNA was synthesized using 500 ng of RNA and the iScript Select cDNA Synthesis Kit (#170-8897) from BioRad. The qPCRs were performed utilizing SYBR Green on the BioRad C1000 Thermal Cycler and CFX96 Real-Time System, by using SosoFast EvaGreen Supermix (#172-5201) from BioRad. Gradient PCRs were run for each primer to determine appropriate and optimal annealing temperatures. All qPCRs were run in triplicate, using 2 min at 95°C; 40 cycles of 5 sec at 95°C, 5 sec at primer specific temperature. Efficiency standard curves for the primers were generated using serial dilutions, and after all the qPCR cycling, melt curve analysis was conducted using the optimal parameters for the BioRad C1000 thermal cycler (65°C to 95°C, increments of 0.5°C).

Forward and reverse primers for the genes of interest were designed using the PrimerQuest program from Integrated DNA Technologies, and the custom oligos were ordered from Eurofins Genomics. Based on Hvid et al. 2011, recommended reference genes were ordered from Eurofins Genomics with the same forward and reverse primer sequences. The best combination of two reference genes, ATP5b and Sdha, was found using the programs NormFinder (http://moma.dk/normfinder-software) and qbaseplus (Biogazelle), with the stability values meeting the geNorm stability cut offs (CV < 0.25, M-Value < 0.5).

### Statistical analyses

Read aligning to features were counted using htseq-count script from HTSeq-0.5.3p3 python framework. Matrix of raw count was generated in R using edgeR bioconductor package [42, 43]. Raw counts were loaded into DESeq2 bioconductor package [44]. The data was normalized and variance stabilized using statistical procedures implemented in DESeq2. Normalized and variance stabilized data was loaded into arrayQualityMetrics package for further exploratory analysis and outlier detection [45]. Differentially expressed genes were detected using DESeq2 as described in the package manual. Pathway analysis was performed using Generally Applicable Gene-set Enrichment analysis implemented in gage Bioconductor package [46]. And, alternatively, gene set enrichment analysis was done using GOstats Bioconductor package in order to detect over-represented GO categories and KEGG pathways within the list of differentially expressed genes [47]. Multiple comparisons adjustment was performed using Benjamini-Hochberg procedure, and genes with an adjusted p-value below 0.1 were considered differentially expressed [48]. The results are expressed as heat maps and MA plots. For the qRT-PCR data, Student's t-test was used for independent variance to determine significance (p<0.05). Statistical analyses and plotting of the data was performed using MS Excel software for Windows. The results are presented as mean relative expression values ± standard error of the mean (SEM).
